# Early Prediction of Cerebral Vasospasm After Aneurysmal Subarachnoid Hemorrhage Using a Machine Learning Model and Interactive Web Application

**DOI:** 10.3390/brainsci15111187

**Published:** 2025-10-31

**Authors:** Maria Gollwitzer, Vanessa Mazanec, Markus Steindl, Baran Atli, Nico Stroh-Holly, Anna Hauser, Gracija Sardi, Tobias Rossmann, Stefan Aspalter, Philip Rauch, Eva Horner, Michael Sonnberger, Andreas Gruber, Matthias Gmeiner

**Affiliations:** 1 Department of Neurosurgery, Kepler University Hospital, 4020 Linz, Austria; 2 Medical Faculty, Johannes Kepler University Linz, 4020 Linz, Austria; 3 RISC Software GmbH, 4232 Hagenberg, Austria; 4 Institute of Neuroradiology, Kepler University Hospital Linz, 4020 Linz, Austria; 5 Clinical Research Institute for Neuroscience, Johannes Kepler University Linz, 4020 Linz, Austria

**Keywords:** aneurysmal subarachnoid hemorrhage, cerebral vasospasm, machine learning, risk stratification, predictive modeling, clinical decision support

## Abstract

**Background:** Cerebral vasospasm is a frequent and severe complication after aneurysmal subarachnoid hemorrhage (aSAH), often causing delayed cerebral ischemia (DCI) and poor outcomes. Despite progress in neurocritical care, early vasospasm prediction after aSAH remains challenging due to its multifactorial nature but is essential for timely intervention. **Methods:** We retrospectively analyzed 503 consecutive patients with spontaneous subarachnoid hemorrhage (SAH) treated between 2013 and 2018. Of these, 345 with angiographically confirmed aSAH were included in the primary analysis, and 158 SAH cases in a sensitivity analysis. We extracted demographic, clinical, and imaging parameters including age, sex, Hunt and Hess grade, Fisher scale, aneurysm and treatment features, external ventricular drainage (EVD), and central nervous system (CNS) infection. Seven supervised machine learning (ML) models, including logistic regression and gradient-boosted trees, were trained using nested cross-validation and evaluated by AUC-ROC, AUC-PR, accuracy, precision, sensitivity, specificity, and F1 score. **Results:** Over half of aSAH patients developed moderate to severe vasospasm. Independent predictors included younger age, higher Hunt and Hess and Fisher grades, and EVD placement (all *p* < 0.001). Logistic regression achieved the best discrimination (AUC-ROC 0.723), while tree-based models reached higher sensitivity (0.867) at the expense of specificity. Aneurysmal etiology further increased vasospasm risk (OR 4.72). **Conclusions:** Routinely available clinical and imaging parameters enable reliable ML-based vasospasm prediction after aSAH. Logistic regression provided the best balance between accuracy and interpretability, while tree-based models optimized sensitivity. This web-based, interpretable ML tool—one of the first using routine clinical data—may support the bedside prediction of vasospasm and requires prospective validation.

## 1. Introduction

Aneurysmal subarachnoid hemorrhage (aSAH) represents a significant burden on healthcare systems worldwide [1]. Despite substantial advances in the management of ruptured intracranial aneurysms, aSAH remains a devastating neurological condition, with an annual incidence of approximately 6–9 cases per 100,000 individuals and a global prevalence of approximately 8 million cases [1,2,3]. The severity of the disease is reflected in the fact that it ranks 36th among the leading causes of death worldwide and 59th among the leading causes of disability-adjusted life years (DALYs) [4]. While early aneurysm repair by surgical clipping or endovascular coiling has markedly improved survival, secondary complications remain common and account for much of the long-term disability [5,6]. One of the most severe is cerebral vasospasm, which typically develops between days 3 and 14 after the initial hemorrhage and frequently results in DCI and permanent neurological deficit [5]. Vasospasm and DCI are recognized as the most important potentially preventable causes of poor outcome after aSAH and contributes substantially to prolonged intensive care unit stay, increased healthcare costs, and impaired quality of life [4,5,7]. The pathophysiology of cerebral vasospasm is complex and multifactorial, involving exposure of cerebral arteries to blood breakdown products, endothelial dysfunction, inflammation, and microvascular dysregulation [5,8,9,10].

Early and reliable identification of patients at risk for vasospasm is essential, as timely targeted interventions—such as hemodynamic augmentation, close neuromonitoring, or endovascular rescue therapy—may mitigate the risk of irreversible ischemic damage [11]. However, in routine clinical practice, prediction remains challenging [12]. Traditional risk stratification relies primarily on clinical grading systems such as the Hunt and Hess or World Federation of Neurological Societies (WFNS) scales, in combination with radiological markers like the Fisher grade [2,5,13]. While these parameters are useful, they often lack sufficient predictive accuracy, especially when applied to heterogeneous patient populations with diverse clinical courses [14].

Artificial intelligence (AI) refers to computational systems capable of performing tasks that traditionally require human cognitive functions, such as learning, reasoning, and decision-making [15]. Within medicine, AI encompasses a broad range of techniques—including rule-based systems, computer vision, and, more recently, machine learning (ML)—that enable data-driven pattern recognition and predictive modeling [16]. While early attempts to automate diagnostics and clinical forecasting using conventional computer methods were limited by data scarcity and processing constraints, recent advances in computational power, data availability, and algorithmic design (e.g., deep learning architectures) have made AI-based approaches both feasible and increasingly accurate [15,17]. Consequently, AI has become an essential component of modern clinical research, offering the potential to uncover complex, nonlinear associations beyond the reach of traditional statistical models [18].

In recent years, ML has emerged as a promising approach for individualized prediction in complex clinical settings [19]. The ML algorithms are capable of processing high-dimensional, multimodal clinical data and uncovering nonlinear relationships beyond the scope of conventional regression models [19,20]. Preliminary studies have demonstrated the feasibility of ML-based prediction of vasospasm and DCI, but most have been limited by small sample sizes, methodological shortcomings or lack of external validation [12,21,22,23].

Recent work between 2023 and 2025 has revisited vasospasm and delayed cerebral ischemia (DCI) prediction after aneurysmal subarachnoid hemorrhage using both classical clinical scores and modern machine-learning (ML) approaches [21,24,25,26,27,28]. Despite encouraging results, the predictive performance and generalizability of these models remain inconsistent across centers and populations. Major challenges include the limited discriminative power of conventional grading systems, heterogeneous outcome definitions (angiographic vasospasm vs. DCI), and high variability in imaging-based assessment methods (CTA, MRA, DSA, and TCD) [21,25,26]. Furthermore, most existing studies are based on single-center datasets with moderate size, class imbalance, and insufficient external validation [24,27]. Explainability, calibration, and reproducibility also remain underreported, complicating clinical adoption [25,27]. These constraints highlight the urgent need for interpretable ML models trained on routinely collected clinical and imaging data, validated through rigorous nested cross-validation, and designed to support early, bedside-level vasospasm prediction.

In this context, the present study aimed to develop and validate a supervised ML-based model for the early prediction of clinically significant cerebral vasospasm following aSAH. Routinely collected demographic, clinical, and radiological variables, including severity scores, aneurysm characteristics, EVD and treatment modality were incorporated into model training. To ensure robust and unbiased performance estimation, we employed rigorous nested cross-validation. The resulting model was subsequently implemented into an interactive web application designed for clinician-oriented use at the bedside.

We hypothesized that ML models, particularly interpretable algorithms such as logistic regression, would enable robust and clinically meaningful vasospasm prediction, thereby facilitating data-driven risk stratification, earlier intervention and ultimately improved outcomes for patients suffering from aSAH.

## 2. Materials and Methods

A retrospective analysis was conducted of all patients diagnosed with spontaneous subarachnoidal haemorrhage (SAH) at the Department of Neurosurgery, Kepler University Hospital, between January 2013 and December 2018. In total, 503 consecutive patients were identified. After diagnostic work-up including digital subtraction angiography, 345 patients were confirmed to have aSAH, while in 158 patients no vascular source was detected. The primary analysis was restricted to patients with aSAH (n = 345), given their relatively homogenous pathophysiology and the well-established clinical relevance of cerebral vasospasm in this population. Inclusion criteria comprised radiological or intraoperative confirmation of a ruptured intracranial aneurysm and complete documentation of relevant clinical and radiological parameters. Patients with traumatic subarachnoidal haemorrhage, non-aneurysmal vascular malformations, or incomplete records were excluded. Relevant demographical and clinical variables were extracted from electronic medical records and all radiological variables were extracted and adjudicated by a board-certified neuroradiologist (co-author, MS). To minimize potential sources of bias inherent in retrospective data collection, all clinical and radiological variables were independently verified by two investigators. Data entries extracted from the electronic health record were double-checked for completeness, plausibility, and internal consistency. In cases of discrepancy, the original medical records and imaging reports were re-examined to ensure accuracy. As this study was a retrospective single-center analysis based on routinely collected data, a formal risk-of-bias assessment tool was not applicable; however, the described verification procedure served to enhance data integrity and methodological transparency. These included age at diagnosis, sex, Hunt and Hess grade at admission, Fisher scale, presence of CNS infection during treatment, use of EVD, aneurysm presence, aneurysm location, aneurysm maximum diameter and dome height, treatment modality (surgical clipping or endovascular coiling), and occurrence and severity of cerebral vasospasm. In addition, a secondary sensitivity analysis was performed on the entire SAH cohort (n = 503), including both aneurysmal and non-aneurysmal cases. This analysis served to assess the generalizability of the predictive models in a broader, real-world clinical setting and to explore whether inclusion of SAH substantially influenced performance metrics. For anonymization, all data were pseudonymized by removing direct identifiers, rounding age to integer values, and randomly shuffling the order of entries. Rows were re-enumerated from 1 to 503 prior to analysis and dataset release. The study was conducted in accordance with the Declaration of Helsinki and was approved by the Ethics Committee of the State of Upper Austria (protocol code: 1028/2019, 27 March 2019). The committee has since been renamed the Ethics Committee of the JKU Faculty of Medicine.

### 2.1. Definition of Cerebral Vasospasm

Cerebral vasospasm was defined as angiographic narrowing of intracranial arteries occurring within 14 days after aSAH [13,29,30]. In clinical practice, assessment is typically performed using computed tomography angiography (CTA), magnetic resonance angiography (MRA), or digital subtraction angiography (DSA), depending on institutional standards [5]. Severity was graded as none (no narrowing), mild (≤1/3), moderate (>1/3–≤2/3), or severe (>2/3 luminal narrowing) [31,32]. For distal vessels, narrowing was only counted if at least two branches were affected [31,33]. For global assessment, mild vasospasm was placed into the none category, resulting in three groups: none, moderate, and severe. In this scheme, moderate vasospasm was defined as either ≥2 vessel segments with moderate narrowing or one segment with severe narrowing, whereas severe vasospasm requires ≥2 segments with severe narrowing. In addition to angiographic imaging, daily transcranial Doppler (TCD) monitoring was also used to support diagnosis [1]. In our study, “daily” transcranial Doppler (TCD) referred to routine bedside examinations performed once per day, not continuous 24 h monitoring. The examinations were conducted using standard TCD probes without the use of a monitoring helmet. Measurements included bilateral assessment of the middle cerebral and internal carotid arteries, evaluating mean flow velocities and Lindegaard ratios according to established clinical practice. Mean middle cerebral artery (MCA) flow velocities of 120–200 cm/s or daily increase >50 cm/s indicated moderate vasospasm, while velocities >200 cm/s were considered severe. These TCD thresholds are widely applied to complement the radiological and hemodynamic assessment and increase diagnostic accuracy [32,34,35]. Management followed institutional protocols, including hemodynamic augmentation to maintain cerebral perfusion and endovascular rescue therapy when indicated [1,2,6,13,30,33].

### 2.2. Features and Data Preprocessing

A comprehensive set of clinical and radiological features was extracted to develop the dataset for predicting the occurrence of cerebral vasospasm in patients with aSAH. Demographic variables included age at diagnosis and sex, along with established grading systems such as the Hunt and Hess score and the Fisher scale to reflect aSAH severity and initial risk. Relevant medical complications, particularly CNS infections, were also documented.

Aneurysm-specific characteristics, including location, maximum diameter, and dome height, were added to provide a detailed morphological description. Procedural factors, such as placement of EVD and treatment modality (surgical clipping or endovascular treatment), were included to account for differences in patient management. Occurrence and severity of cerebral vasospasm were systematically recorded. Patients with missing data were excluded. The published dataset consists of 503 patients, of whom 345 had aSAH and 158 had SAH. The primary analysis was restricted to the 345 fully documented aSAH cases.

### 2.3. Statistical Analysis

Normality within vasospasm groups was assessed using the Shapiro–Wilk test. Depending on the outcome, either a two-sample *t*-test or the Mann–Whitney U test was applied for comparisons of metric data. Associations between categorical variables were examined using the Chi-squared test, with Fisher’s exact test applied for 2 × 2 tables when any cell count was less than 5. Statistical significance was defined as a two-sided *p* level of 0.05. Analyses were performed in R version 4.5.1 (R Foundation for Statistical Computing, Vienna, Austria). Tables were produced in R version 4.5.1 with gtsummary.

### 2.4. Predictive Modeling

To predict moderate or severe cerebral vasospasm, a range of supervised machine learning algorithms was implemented. The primary analysis was conducted in patients with aSAH (n = 345), and a secondary sensitivity analysis was performed in the entire cohort of SAH patients (n = 503).

These included logistic regression, decision trees, random forests, gradient boosted trees, linear and nonlinear support vector machines (SVMs), and k-nearest neighbors (KNN). Model development and training were carried out in Python 3.11 using the scikit-learn, NumPy, and pandas libraries.

Starting from the published dataset, additional preprocessing was performed to ensure comparability and optimal model performance. Continuous variables were standardized by centering and scaling to unit variance, and categorical variables were encoded using one-hot encoding. For aneurysm location, categories were consolidated into two clinically relevant groups: anterior communicating artery (ACom) and all other locations.

**Figure 1:**
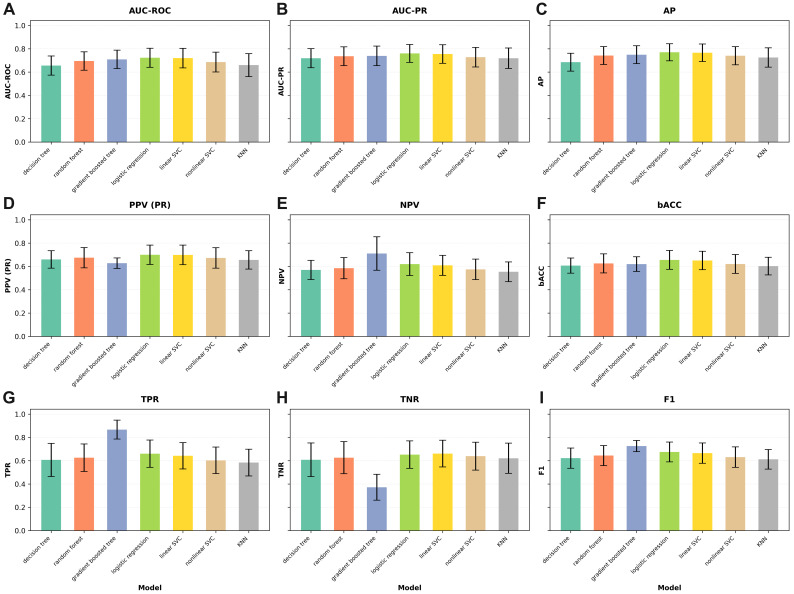
Bar charts illustrating the predictive performance of machine learning models in the aSAH cohort (N = 345) across nine evaluation metrics: (**A**) AUC-ROC, (**B**) AUC-PR, (**C**) AP, (**D**) PPV, (**E**) NPV, (**F**) bACC, (**G**) TPR, (**H**) TNR, and (**I**) F1-score. Each chart shows the mean metric values for all models, enabling visual comparison of their relative performance.

The predictive performance of the models in the aSAH cohort is presented in Table 1 below, whereas Table 2 summarizes the results for the entire cohort. Figure 1 below provides a visual representation of the predictive performance of the machine learning models in the aSAH cohort (N = 345) across nine evaluation metrics.

Imbalance in the outcome distribution was addressed by applying class weights inversely proportional to class frequencies for all models except KNN. For the KNN classifier, the Synthetic Minority Oversampling Technique (SMOTE) was used to balance the minority class.

Model training followed a repeated nested cross-validation (CV) procedure. The outer loop used 10-fold CV with 10 repetitions to obtain stable performance estimates. At each outer iteration, nine folds were used for training and one for testing. Within the training portion, a 10-fold inner CV was performed for hyperparameter optimization, with grid search applied for simpler models and randomized search for more complex models. The best hyperparameter set was selected according to balanced accuracy, and the resulting model was evaluated on the held-out outer test fold.

Discriminative performance was assessed using multiple metrics, including the area under the receiver operating characteristic curve (AUC-ROC), the area under the precision–recall curve (AUC-PR), average precision (AP), balanced accuracy (bACC), precision (positive predictive value, PPV), negative predictive value (NPV), sensitivity (true positive rate, TPR), specificity (true negative rate, TNR), and the F1 score. All metrics were reported as mean and standard deviation across the test folds.

**Figure 2:**
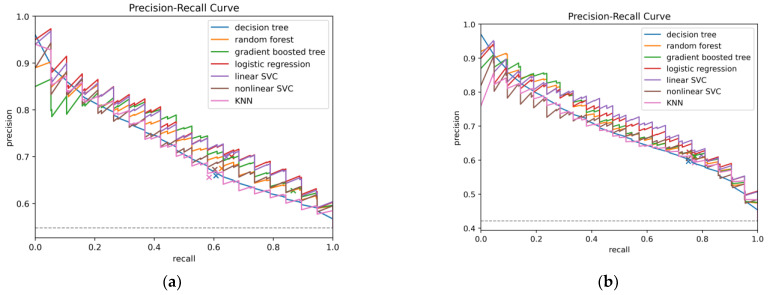
Precision–recall curves for all machine learning models predicting moderate or severe cerebral vasospasm: (**a**) in patients with aSAH; (**b**) in the full cohort. Curves were mean-aggregated across all test folds of repeated cross-validation, with the horizontal line indicating baseline precision. For each model, the point corresponding to the default decision threshold is indicated.

**Figure 3:**
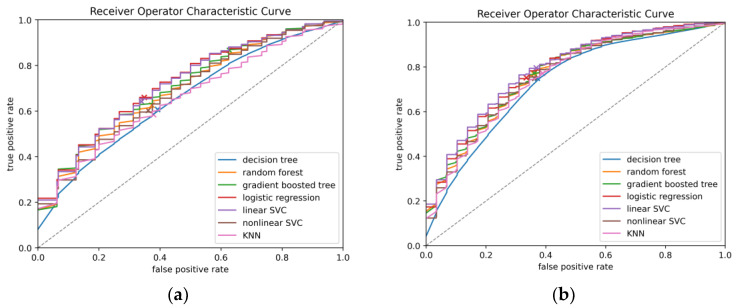
Receiver operating characteristic (ROC) curves for all machine learning models: (**a**) in patients with aSAH; (**b**) in the full cohort, predicting moderate or severe cerebral vasospasm. Curves were mean-aggregated across all test folds of repeated cross-validation, with the diagonal line representing baseline performance. For each model, the point corresponding to the default decision threshold is indicated.

To illustrate model performance, predictions from all test folds were mean-aggregated to construct ROC and precision–recall (PR) curves (Figure 2 and Figure 3).

These curves depict the trade-offs between true positive rate (TPR) and false positive rate (FPR) for ROC, and between precision (PPV) and recall (sensitivity) for PR. Finally, the contribution of individual predictors in the logistic regression model was evaluated in two ways: odds ratios derived from unscaled coefficients were reported for clinical interpretability, while standardized coefficients from the scaled model were examined separately to compare the relative importance of predictors.

### 2.5. Data and Code Availability

In this study, both the dataset and the source code used to develop the machine learning models are made freely available to the public. The dataset is provided under a Creative Commons Attribution (CC BY) license, and the source code is released under the Massachusetts Institute of Technology (MIT) license. Both resources can be accessed at https://github.com/RISCSoftware/vasospasm-prediction (accessed on 1 October 2025) Making these materials publicly available supports the reproducibility of our findings and encourages their integration and further development in future research.

### 2.6. Web-Based Risk Estimation Interface

To support practical application, an interactive web-based interface was developed. This tool, implemented using Gradio (https://www.gradio.app/, accessed on 1 October 2025) and hosted at https://huggingface.co/spaces/risc42/vasospasm-prediction, (accessed on 1 October 2025) allows users to enter patient-specific data and receive predictions on the probability of developing moderate or severe cerebral vasospasm. In contrast to the models evaluated using nested cross-validation for robust performance estimation, the deployed version was trained on the entire dataset to optimize predictive accuracy for real-time use.

The Web application loads pre-trained scikit-learn models from disk, processes the input data provided by the user, and generates a binary prediction indicating the occurrence of vasospasm (yes or no). Where appropriate, the estimated probability and odds ratio are also displayed, providing additional context for interpretation. This web-based implementation is intended to promote the clinical transferability of our model and support informed decision-making in everyday practice.

## 3. Results

Of the 345 patients with aSAH included in the primary analysis, 189 (55%) developed moderate or severe cerebral vasospasm during their hospital stay. Overall, 236 (68%) were female and 109 (32%) male. Most ruptured aneurysms were located in the anterior circulation (87%), with anterior communicating artery (ACom, n = 120) and middle cerebral artery (MCA, n = 69) being the largest subgroups. The majority were treated endovascularly with coiling (83%), while 14% underwent microsurgical clipping. During treatment, 151 patients (44%) required EVD, and 32 (9.3%) developed CNS infection. A detailed summary of baseline characteristics is provided in Table 3.

The EVD placement was significantly more frequent among patients with vasospasm compared to those without (*p* = 0.001). By contrast, CNS infection was not associated with vasospasm occurrence (*p* = 0.5). Younger age, higher Hunt and Hess grade, and higher Fisher score at admission were strongly associated with vasospasm (all *p* < 0.001), whereas aneurysm location (*p* = 0.2) and treatment modality (*p* = 0.6) showed no significant influence (Table 3).

In the secondary analysis, all 503 patients with spontaneous SAH, 212 (≈42.1%) developed moderate or severe vasospasm. Overall, 345 (68.6%) had aSAH and 158 (31.4%) SAH. Overall, 184 (37%) of patients required EVD implantation and 40 (8%) developed CNS infection. Univariate analysis demonstrated that younger age, aneurysmal location, EVD placement, higher Hunt and Hess grade and Fisher score were strongly associated with vasospasm (all *p* < 0.001). By contrast, gender and CNS infection showed no significant associations. The overall results were therefore consistent with the primary analysis in the aneurysmal subgroup. A detailed overview of patient characteristics and model metrics is provided in Table 4.

### 3.1. Prediction of Cerebral Vasospasm

In our comparative analysis of ML models to predict moderate or severe cerebral vasospasm in patients with aSAH, logistic regression achieved the best overall discrimination, with an AUC-ROC of 0.723 and an AUC-PR of 0.760. It also reached the highest average precision (0.770) with a balanced accuracy of 0.656, indicating a relatively strong balance between sensitivity and specificity. The gradient-boosted tree model demonstrated the highest sensitivity (TPR 0.867), but this came at the expense of specificity (TNR 0.372), resulting in lower overall balance despite a competitive AUC-ROC (0.709) and AUC-PR (0.739). Random forests showed stable performance (AUC-ROC 0.695, AUC-PR 0.736) with a moderate trade-off between sensitivity (0.626) and specificity (0.626). The support vector classifiers (SVCs) performed comparably to logistic regression in terms of discrimination. The linear SVC reached an AUC-ROC of 0.720, an AUC-PR of 0.755, and a balanced accuracy of 0.651, performing second-best across these metrics. The nonlinear SVC achieved slightly lower performance (AUC-ROC 0.686, AUC-PR 0.728) but maintained a reasonable balance between sensitivity (0.603) and specificity (0.639). The decision tree model performed weakest overall (AUC-ROC 0.656), though it maintained a F1 score of 0.622 and moderate sensitivity (0.607). The k-nearest neighbors (KNN) classifier yielded the second lowest discrimination (AUC-ROC 0.660, AUC-PR 0.719) and balanced accuracy (0.603) but maintained a specificity (0.621) similar to decision trees and random forests. Across all models, the negative predictive value (NPV) remained consistently moderate (0.554–0.711), reflecting a reliable ability to exclude patients unlikely to develop vasospasm. The positive predictive value (PPV) was modest (0.628–0.700), underscoring the inherent challenge of identifying true positives in this setting. Overall, based on AUC metrics, balanced accuracy, and F1 score, logistic regression emerged as the most robust and clinically interpretable model. Detailed results including all performance metrics are provided in Table 1.

In the sensitivity analysis with all 503 patients, the overall performance of the models improved compared to the aneurysmal subgroup. Logistic regression again ranked among the best-performing algorithms, achieving an AUC-ROC of 0.786, an AUC-PR of 0.717, an average precision of 0.728, and an F1 score of 0.679. Its balanced accuracy was 0.709, with a sensitivity of 0.752 and specificity of 0.666, reflecting a strong overall trade-off between sensitivity and specificity. The linear support vector classifier (SVC) yielded the highest discrimination overall (AUC-ROC 0.791, AUC-PR 0.727) and reached the best F1 score (0.692), highlighting its robust performance in the full cohort. Gradient-boosted trees and random forests also performed competitively (AUC-ROC 0.769 and 0.764, respectively) with good balance between sensitivity and specificity. The decision tree achieved the lowest overall discrimination (AUC-ROC 0.734), balanced accuracy (0.689), and F1 score (0.659), while the nonlinear SVC and k-nearest neighbors (KNN) models showed intermediate performance with AUC-ROC values of 0.762 and 0.759, respectively. Across all models, the negative predictive value (NPV) remained consistently high (0.784–0.814), supporting their reliability in ruling out patients unlikely to develop vasospasm. The positive predictive value (PPV) was modest (range 0.592–0.624), reflecting the challenge of accurately identifying true positives. Taken together, these results indicate that both logistic regression and linear SVC achieved the most stable and balanced performance in the full cohort, while tree-based models provided complementary strengths in sensitivity. Detailed results are summarized in Table 2.

### 3.2. Feature Importance in Logistic Regression

Analysis of logistic regression odds ratios and standardized coefficients identified several independent predictors of cerebral vasospasm in the aSAH cohort (n = 345). Younger age was strongly associated with increased vasospasm risk (OR 0.95 ± 0.02), indicating a protective effect of older age. Higher Hunt and Hess grade (OR 1.27 ± 0.12) and higher Fisher score (OR 1.38 ± 0.18) were both strongly associated with vasospasm occurrence. In addition, the requirement for EVD emerged as an independent risk factor (OR 1.48 ± 0.22). By contrast, CNS infection (OR 1.15 ± 0.14), aneurysm size parameters (diameter OR 0.96 ± 0.03, height OR 1.00 ± 0.01), sex (OR 1.11 ± 0.12), and treatment modality (clipping OR 0.93 ± 0.14, coiling OR 0.83 ± 0.13) showed only weak, non-significant associations. Similarly, aneurysm location in the anterior communicating artery was associated with a lower odds ratio (OR 0.64 ± 0.14), though this effect appeared weak. These findings confirm that age, clinical severity (Hunt and Hess), radiological blood load (Fisher), and EVD placement are the strongest independent predictors of vasospasm in the aSAH cohort, while other variables such as aneurysm morphology, treatment modality, and sex exerted minimal influence. A detailed overview of regression coefficients and odds ratios is provided in Table 5.

In the full cohort of 503 patients with spontaneous SAH, logistic regression identified younger age, higher Hunt and Hess grade, higher Fisher score, and the requirement for EVD as independent predictors of cerebral vasospasm. Specifically, each additional year of age was associated with a reduced risk of vasospasm (OR 0.96 ± 0.02), while higher Hunt and Hess grade (OR 1.25 ± 0.11) and Fisher score (OR 1.38 ± 0.18) were associated with increased odds. EVD placement was also a robust predictor (OR 1.57 ± 0.25). The presence of an aneurysmal source of hemorrhage was strongly associated with vasospasm (OR 4.72 ± 3.80), further underscoring the higher risk profile of aSAH compared to SAH, while treatment modality (clipping OR 1.42 ± 0.37, coiling OR 1.36 ± 0.32) showed modestly elevated odds ratios but with wide variability. By contrast, CNS infection (OR 1.01 ± 0.11), aneurysm morphology (diameter OR 0.98 ± 0.03, height OR 1.02 ± 0.02), and sex (OR 1.01 ± 0.06) showed odds ratios close to 1, indicating minimal influence on vasospasm risk. Aneurysm location in the anterior communicating artery was associated with a lower risk (OR 0.69 ± 0.18). These findings indicate that, even in the broader SAH population, younger age, clinical severity (Hunt and Hess), radiological blood burden (Fisher), EVD placement, and aneurysmal etiology are the most relevant predictors of cerebral vasospasm. Full details of regression coefficients and odds ratios are provided in Table 6.

## 4. Discussion

In this retrospective single-center analysis of 345 patients with aSAH, we demonstrated that younger age, higher Hunt and Hess grade, higher Fisher score, and the placement of an EVD were independent predictors of developing moderate to severe cerebral vasospasm. Based on these findings, we developed several ML models, among which logistic regression achieved the most favorable balance between sensitivity, specificity, and interpretability. Gradient-boosted trees yielded higher sensitivities but at the expense of substantially reduced specificity. This trade-off is clinically relevant, as high sensitivity is crucial for the early identification of patients at risk, whereas minimizing false-positive predictions is equally important to avoid overdiagnosis and unnecessary interventions.

These predictors are pathophysiologically plausible and consistent with current understanding of vasospasm after aSAH. The Fisher score reflects the burden of subarachnoid blood, exposing cerebral arteries to hemoglobin degradation products that trigger oxidative stress, endothelin release, endothelial dysfunction, and inflammatory cascades [5,8,9,10,31,32,33].

The pathophysiological cascade following subarachnoid hemorrhage is strongly influenced by oxidative and nitrosative stress. The breakdown of hemoglobin releases free heme and iron, which catalyze the formation of reactive oxygen species (ROS) and reactive nitrogen species (RNS), resulting in lipid peroxidation, protein oxidation, and DNA damage [36,37]. In addition, oxidative stress disrupts endothelial nitric oxide synthase (eNOS) activity, leading to impaired nitric oxide (NO) bioavailability and endothelial dysfunction [38]. This imbalance promotes cerebral vasoconstriction and microvascular dysregulation, aggravating ischemic injury. Excitotoxicity mechanisms are also triggered, with excessive glutamate release and NMDA receptor overactivation contributing to neuronal calcium overload and mitochondrial dysfunction [39]. Recent studies further highlight that SAH induces redox-sensitive signaling alterations, including activation of NF-κB and MAPK pathways, which amplify inflammation and vascular injury [36,40]. These combined oxidative and inflammatory processes contribute critically to the development of cerebral vasospasm and delayed cerebral ischemia.

Higher Hunt and Hess grade at admission indicates severe initial injury, which correlates with extensive bleeding, raised intracranial pressure, and secondary ischemic mechanisms, all of which predispose to vasospasm [2,5,13]. The requirement for EVD placement should not be interpreted as an independent causal risk factor but rather as a surrogate marker of overall disease severity. Its indication generally reflects the presence of extensive intraventricular hemorrhage, hydrocephalus, and elevated intracranial pressure—conditions that have themselves been consistently associated with an increased likelihood of vasospasm [29,30,33]. Accordingly, EVD serves primarily as an indirect indicator of hemorrhage burden and clinical complexity rather than as a direct contributor to the underlying pathophysiology. This interpretation aligns with recent pragmatic reviews on vasospasm management, which similarly emphasize the role of EVD as a marker of severe clinical courses rather than as an independent etiological factor [29,30,33]. Finally, younger age was strongly associated with vasospasm, corroborating reports of increased vascular reactivity and reduced protective effects of cerebral atrophy in older patients [41]. The additional identification of aneurysmal etiology in the full cohort underscores the higher vasospasm risk in aSAH compared to perimesencephalic hemorrhage, which typically follows a benign course [42,43].

Traditional grading systems (Hunt and Hess, WFNS) and radiological scores (Fisher) remain the clinical backbone of risk assessment [2,5,13], yet their stand-alone predictive accuracy is limited and heterogenous across populations [14,44]. Our findings echo Harrod et al.’s conclusion that blood burden is the most consistent predictor [44] while demonstrating that routinely available clinical variables—Hunt and Hess grade, age, and EVD—add independent predictive information. This supports guideline-endorsed monitoring strategies that scale with hemorrhage burden and clinical severity [1,5,6,29].

The use of ML models in our study demonstrates that robust predictions can already be achieved using routinely collected clinical variables. Logistic regression provided the best balance between sensitivity and specificity, whereas tree-based models (gradient-boosted trees) achieved higher sensitivity but at the cost of reduced specificity. This trade-off is clinically relevant, while high sensitivity is desirable to capture as many at-risk patients as possible at an early stage, the rate of false-positive predictions must be minimized to avoid unnecessary invasive interventions and resource utilization.

Our findings complement previous ML-based studies, most of which relied on small cohorts and heterogeneous methodologies [12,21,22,23]. For instance, Zarrin et al. [21] demonstrated relevant improvements in vasospasm prediction using ML approaches, while a recent meta-analysis by Zhang et al. [22] confirmed the overall suitability of ML methods but also highlighted methodological limitations and the lack of external validation. Ramos et al. [45] already showed in 2019 that ML-based models can improve the prediction of DCI. Our study adds to this growing field by analyzing a larger cohort (n = 503) and applying rigorous nested cross-validation to minimize overfitting.

Within the emerging ML literature, our work complements several trajectories. Kim et al. used explainable modeling in 343 aSAH patients and highlighted age and aneurysm size alongside Fisher and clinical severity [46]. After multivariable adjustment, we did not observe independent effects of aneurysm morphology, echoing studies in which morphology attenuates when severity and blood load are accounted for [44,46]. Beyond vasospasm per se, Ramos et al. demonstrated that multimodal models incorporating imaging-derived features improve DCI prediction compared with clinical variables alone, albeit with reduced transparency [45]. Systematic and meta-analytic appraisals similarly suggest that ML can enhance risk prediction for vasospasm and DCI, but frequently note small samples, heterogeneous endpoints, and limited external validation [12,21,22,23]. Against this backdrop, our deliberate choice of interpretable models trained on routine variables—evaluated with repeated nested cross-validation—prioritizes generalizability, reproducibility, and immediate bedside applicability without dependence on specialized radiomics pipelines.

Although several algorithms achieved reasonable discrimination, logistic regression offered the most favorable balance between accuracy and interpretability. Tree-based models achieved higher sensitivity but lower specificity, raising concerns about false positives and unnecessary interventions [6,11,30]. Logistic regression provides clear odds ratios, which clinicians can interpret within established clinical reasoning frameworks [19,20]. Embedding such models into interactive web-based interfaces may further support bedside decision-making, facilitating early identification of high-risk patients, intensified monitoring, and timely therapeutic intervention.

Interpretability is a key consideration. While more complex models such as random forests or gradient boosted trees can yield higher sensitivities, they are often regarded as “black boxes.” In contrast, logistic regression provides transparent odds ratios that can be directly interpreted in the clinical context—an essential factor for acceptance in routine practice [19,20].

The implementation of such models into web-based applications could enhance clinical workflows, facilitate early interventions, and thereby reduce morbidity and mortality after aSAH in the long term.

### Limitations and Future Directions

This study has several limitations. First, its retrospective, single-center design may limit generalizability, and external validation in multicenter cohorts is necessary. Furthermore, the analysis was restricted to in-hospital data up to the occurrence of vasospasm or discharge. No long-term follow-up data were available for inclusion, which precluded assessment of delayed complications, neurological recovery, or long-term outcomes. Future research should therefore include prospective longitudinal follow-up to evaluate the sustained clinical impact and predictive validity of ML-based vasospasm models. Second, we relied exclusively on clinical and radiological variables available in routine care; integration of advanced imaging features, laboratory markers, or perfusion data could further improve predictive accuracy. In addition, our dataset did not allow for a reliable differentiation of infection origin (e.g., procedure-related versus hematogenic). Central nervous system infections were recorded as a binary variable based on clinical diagnosis and microbiological confirmation, without detailed etiological classification. Another limitation concerns the absence of intracranial pressure (ICP) monitoring data in the predictive modeling. Although ICP was continuously measured in patients with an external ventricular drain (EVD), these recordings were not consistently available across the full cohort due to differences in monitoring duration, data storage, and completeness of electronic documentation. To minimize bias from missing data, ICP was therefore not included as a predictive feature in the machine learning models. Nevertheless, ICP dynamics are physiologically linked to vasospasm pathogenesis through mechanisms of reduced cerebral perfusion pressure, microcirculatory compromise, and impaired autoregulation. Future studies should aim to incorporate continuous physiological parameters—such as ICP, cerebral perfusion pressure, and multimodal neuromonitoring data—into ML frameworks to improve early prediction and clinical applicability. Third, while performance metrics were consistent across both cohorts, the achieved AUCs remained moderate, indicating that vasospasm prediction remains challenging.

Future research should therefore focus on prospective validation in larger, heterogeneous populations, exploration of multimodal feature sets including imaging and biomarkers, and continuous updating of predictive models. Ultimately, integrating interpretable ML tools into clinician-friendly interfaces holds promise to improve vasospasm risk stratification, optimize monitoring strategies, and reduce the incidence of DCI in patients with aSAH [47].

## 5. Conclusions

In this retrospective single-center study, we identified younger age, higher Hunt and Hess grade, higher Fisher score, and the need for EVD as robust and pathophysiologically plausible predictors of moderate to severe vasospasm after aSAH. Building on these findings, we developed and validated ML models using routinely available clinical variables. Logistic regression achieved the most favorable balance between accuracy, interpretability, and robustness, while more complex tree-based models provided higher sensitivity at the expense of reduced specificity. This underscores the trade-off between performance and interpretability that is central to clinical implementation.

Our results complement previous ML-based research by demonstrating that reliable risk prediction is achievable without reliance on advanced imaging or radiomics, thereby enhancing generalizability and bedside applicability. Future work should focus on multicenter, prospective validation of the model, integration of multimodal data such as perfusion imaging and biomarkers, and the development of continuously learning systems that update model performance over time. Furthermore, usability testing of the web-based interface in real clinical workflows will be essential to assess its impact on decision-making and patient outcomes.

Integrating such interpretable, data-driven tools into clinician-friendly, web-based platforms holds promise to facilitate early identification of high-risk patients, support tailored monitoring strategies, and enable timely therapeutic interventions. Ultimately, these efforts may contribute to reducing the incidence of delayed cerebral ischemia and improving neurological recovery after aSAH.

Future Work Roadmap:

1. Multicenter prospective validation: Confirm the model’s generalizability in larger, independent patient cohorts across multiple institutions.
2. Integration of multimodal data: Incorporate advanced imaging (e.g., perfusion imaging) and biomarker information to further improve predictive performance.
3. Continuous learning systems: Develop dynamic models capable of updating performance as new data become available over time.
4. Clinical workflow integration: Evaluate the usability and impact of the web-based application in real clinical settings.
5. Outcome improvement: Assess whether early vasospasm risk identification leads to optimized monitoring strategies and improved patient outcomes.

## Figures and Tables

**Table 1:** Performance of predictive models in the aSAH cohort (N = 345).

Model	AUC-ROC	AUC-PR	AP	PPV (PR)	NPV	bACC	TPR	TNR	F1
**decision tree**	0.656 ± 0.082	0.719 ± 0.082	0.685 ± 0.077	0.660 ± 0.075	0.570 ± 0.082	0.607 ± 0.065	0.607 ± 0.142	0.608 ± 0.144	0.622 ± 0.087
**random forest**	0.695 ± 0.079	0.736 ± 0.081	0.742 ± 0.077	0.675 ± 0.087	0.585 ± 0.091	0.626 ± 0.082	0.626 ± 0.118	0.626 ± 0.138	0.644 ± 0.086
**gradient boosted tree**	0.709 ± 0.079	0.739 ± 0.084	0.749 ± 0.077	0.628 ± 0.045	0.711 ± 0.144	0.620 ± 0.063	0.867 ± 0.081	0.372 ± 0.111	0.726 ± 0.048
**logistic regression**	0.723 ± 0.082	0.760 ± 0.077	0.770 ± 0.073	0.700 ± 0.083	0.620 ± 0.098	0.656 ± 0.081	0.660 ± 0.117	0.652 ± 0.118	0.675 ± 0.085
**linear SVC**	0.720 ± 0.084	0.755 ± 0.080	0.766 ± 0.075	0.699 ± 0.084	0.609 ± 0.086	0.651 ± 0.079	0.642 ± 0.113	0.661 ± 0.115	0.665 ± 0.087
**nonlinear SVC**	0.686 ± 0.085	0.728 ± 0.084	0.740 ± 0.078	0.673 ± 0.088	0.575 ± 0.088	0.621 ± 0.081	0.603 ± 0.114	0.639 ± 0.120	0.631 ± 0.088
**KNN**	0.660 ± 0.098	0.719 ± 0.088	0.725 ± 0.083	0.656 ± 0.079	0.554 ± 0.085	0.603 ± 0.076	0.584 ± 0.115	0.621 ± 0.130	0.612 ± 0.084

**Table 2:** Performance of predictive models in the overall cohort (N = 503).

Model	AUC-ROC	AUC-PR	AP	PPV (PR)	NPV	bACC	TPR	TNR	F1
**decision tree**	0.734 ± 0.071	0.688 ± 0.083	0.630 ± 0.084	0.596 ± 0.058	0.786 ± 0.082	0.689 ± 0.061	0.750 ± 0.132	0.629 ± 0.087	0.659 ± 0.075
**random forest**	0.764 ± 0.065	0.709 ± 0.083	0.702 ± 0.087	0.611 ± 0.057	0.799 ± 0.072	0.704 ± 0.057	0.769 ± 0.108	0.640 ± 0.083	0.677 ± 0.065
**gradient boosted tree**	0.769 ± 0.062	0.712 ± 0.077	0.710 ± 0.079	0.611 ± 0.056	0.801 ± 0.075	0.706 ± 0.058	0.773 ± 0.106	0.639 ± 0.077	0.680 ± 0.064
**logistic regression**	0.786 ± 0.064	0.717 ± 0.088	0.728 ± 0.082	0.624 ± 0.060	0.791 ± 0.066	0.709 ± 0.056	0.752 ± 0.093	0.666 ± 0.078	0.679 ± 0.061
**linear SVC**	0.791 ± 0.062	0.727 ± 0.088	0.737 ± 0.082	0.615 ± 0.055	0.814 ± 0.068	0.714 ± 0.054	0.796 ± 0.086	0.632 ± 0.079	0.692 ± 0.055
**nonlinear SVC**	0.762 ± 0.059	0.684 ± 0.076	0.698 ± 0.071	0.605 ± 0.060	0.784 ± 0.073	0.693 ± 0.056	0.749 ± 0.108	0.637 ± 0.092	0.665 ± 0.062
**KNN**	0.759 ± 0.063	0.680 ± 0.087	0.685 ± 0.079	0.592 ± 0.057	0.793 ± 0.080	0.691 ± 0.061	0.772 ± 0.108	0.610 ± 0.080	0.667 ± 0.066

**Table 3:** Baseline Patient and Aneurysm Characteristics: aSAH Subgroup.

Characteristic	Overall N = 345 ^1^	Vasospasm: Yes N = 189 ^1^	Vasospasm: No N = 156 ^1^	*p*-Value ^2^
**Age at aSAH**	56 ± 13	52 ± 12	60 ± 13	<0.001
**Sex**				>0.9
Female	236/345 (68%)	130/189 (69%)	106/156 (68%)	
Male	109/345 (32%)	59/189 (31%)	50/156 (32%)	
**Hunt & Hess grade**				0.001
1	77/345 (22%)	30/189 (16%)	47/156 (30%)	
2	113/345 (33%)	58/189 (31%)	55/156 (35%)	
3	69/345 (20%)	41/189 (22%)	28/156 (18%)	
4	69/345 (20%)	50/189 (26%)	19/156 (12%)	
5	17/345 (4.9%)	10/189 (5.3%)	7/156 (4.5%)	
**Fisher grade**				0.001
1	9/345 (2.6%)	5/189 (2.6%)	4/156 (2.6%)	
2	25/345 (7.2%)	5/189 (2.6%)	20/156 (13%)	
3	112/345 (32%)	58/189 (31%)	54/156 (35%)	
4	199/345 (58%)	121/189 (64%)	78/156 (50%)	
**Aneurysm location**				0.2
ACA	35/345 (10%)	16/189 (8.5%)	19/156 (12%)	
ACom	120/345 (35%)	56/189 (30%)	64/156 (41%)	
AICA	1/345 (0.3%)	1/189 (0.5%)	0/156 (0%)	
BA	23/345 (6.7%)	15/189 (7.9%)	8/156 (5.1%)	
ICA	37/345 (11%)	20/189 (11%)	17/156 (11%)	
MCA	69/345 (20%)	42/189 (22%)	27/156 (17%)	
PCA	5/345 (1.4%)	2/189 (1.1%)	3/156 (1.9%)	
PCom	39/345 (11%)	24/189 (13%)	15/156 (9.6%)	
PICA	10/345 (2.9%)	7/189 (3.7%)	3/156 (1.9%)	
SCA	1/345 (0.3%)	1/189 (0.5%)	0/156 (0%)	
VA	5/345 (1.4%)	5/189 (2.6%)	0/156 (0%)	
**Aneurysm circulation**				0.060
Anterior	300/345 (87%)	158/189 (84%)	142/156 (91%)	
Posterior	45/345 (13%)	31/189 (16%)	14/156 (9.0%)	
**Aneurysm maximum diameter (mm)**	6.1 ± 3.5	6.0 ± 3.4	6.1 ± 3.6	>0.9
**Aneurysm dome height (mm)**	4.90 ± 2.99	4.93 ± 3.11	4.87 ± 2.84	0.8
**Treatment for aSAH**				0.6
Clipping	47/345 (14%)	27/189 (14%)	20/156 (13%)	
Coiling	288/345 (83%)	155/189 (82%)	133/156 (85%)	
Untreated	10/345 (2.9%)	7/189 (3.7%)	3/156 (1.9%)	
**EVD implanted**	151/345 (44%)	98/189 (52%)	53/156 (34%)	0.001
**CNS infection during treatment**	32/345 (9.3%)	20/189 (11%)	12/156 (7.7%)	0.5

^1^ Mean ± SD; n/N (%). ^2^ Welch Two Sample *t*-test; Pearson’s Chi-squared test with Yates’ continuity correction; Pearson’s Chi-squared test; Wilcoxon rank sum test with continuity correction.

**Table 4:** Baseline Patient and Aneurysm Characteristics: All Patients.

Characteristic	Overall N = 503 ^1^	Vasospasm: Yes N = 212 ^1^	Vasospasm: No N = 291 ^1^	*p*-Value ^2^
**Age**	55 ± 13	52 ± 12	58 ± 13	<0.001
**Sex**				0.2
Female	311/503 (62%)	139/212 (66%)	172/291 (59%)	
Male	192/503 (38%)	73/212 (34%)	119/291 (41%)	
**aSAH**	345/503 (69%)	189/212 (89%)	156/291 (54%)	<0.001
**Hunt & Hess grade**				<0.001
1	160/503 (32%)	40/212 (19%)	120/291 (41%)	
2	161/503 (32%)	63/212 (30%)	98/291 (34%)	
3	84/503 (17%)	47/212 (22%)	37/291 (13%)	
4	80/503 (16%)	52/212 (25%)	28/291 (9.6%)	
5	18/503 (3.6%)	10/212 (4.7%)	8/291 (2.7%)	
**Fisher grade**				<0.001
1	20/503 (4.0%)	5/212 (2.4%)	15/291 (5.2%)	
2	50/503 (9.9%)	7/212 (3.3%)	43/291 (15%)	
3	195/503 (39%)	71/212 (33%)	124/291 (43%)	
4	238/503 (47%)	129/212 (61%)	109/291 (37%)	
**Aneurysm location**				0.2
ACA	35/345 (10%)	16/189 (8.5%)	19/156 (12%)	
ACom	120/345 (35%)	56/189 (30%)	64/156 (41%)	
AICA	1/345 (0.3%)	1/189 (0.5%)	0/156 (0%)	
BA	23/345 (6.7%)	15/189 (7.9%)	8/156 (5.1%)	
ICA	37/345 (11%)	20/189 (11%)	17/156 (11%)	
MCA	69/345 (20%)	42/189 (22%)	27/156 (17%)	
PCA	5/345 (1.4%)	2/189 (1.1%)	3/156 (1.9%)	
PCom	39/345 (11%)	24/189 (13%)	15/156 (9.6%)	
PICA	10/345 (2.9%)	7/189 (3.7%)	3/156 (1.9%)	
SCA	1/345 (0.3%)	1/189 (0.5%)	0/156 (0%)	
VA	5/345 (1.4%)	5/189 (2.6%)	0/156 (0%)	
**Aneurysm circulation**				0.060
Anterior	300/345 (87%)	158/189 (84%)	142/156 (91%)	
Posterior	45/345 (13%)	31/189 (16%)	14/156 (9.0%)	
**Aneurysm maximum diameter (mm)**	6.1 ± 3.5	6.0 ± 3.4	6.1 ± 3.6	>0.9
**Aneurysm dome height (mm)**	4.90 ± 2.99	4.93 ± 3.11	4.87 ± 2.84	0.8
**Treatment for aSAH**				0.6
Clipping	47/345 (14%)	27/189 (14%)	20/156 (13%)	
Coiling	288/345 (83%)	155/189 (82%)	133/156 (85%)	
Untreated	10/345 (2.9%)	7/189 (3.7%)	3/156 (1.9%)	
**EVD implanted**	184/503 (37%)	107/212 (50%)	77/291 (26%)	<0.001
**CNS infection during treatment**	40/503 (8.0%)	21/212 (9.9%)	19/291 (6.5%)	0.2

^1^ Mean ± SD; n/N (%). ^2^ Welch Two Sample *t*-test; Pearson’s Chi-squared test with Yates’ continuity correction; Pearson’s Chi-squared test; Wilcoxon rank sum test with continuity correction.

**Table 5:** Odds Ratios and Standardized Logistic Regression Coefficients (aSAH cohort). Values are presented as mean ± standard deviation across test folds from repeated cross-validation.

Feature	Odds Ratio	Standardized Coefficient
Age at aSAH	0.951 ± 0.021	−0.659 ± 0.282
Hunt & Hess grade	1.268 ± 0.116	0.275 ± 0.112
Fisher grade	1.379 ± 0.180	0.231 ± 0.101
CNS infection	1.152 ± 0.137	0.039 ± 0.033
EVD implanted	1.480 ± 0.217	0.189 ± 0.077
Aneurysm	1.000 ± 0.000	0.000 ± 0.000
Aneurysm maximum diameter	0.959 ± 0.025	−0.146 ± 0.090
Aneurysm dome height	1.002 ± 0.016	0.005 ± 0.045
Female sex	1.116 ± 0.111	0.049 ± 0.045
ACom location	0.640 ± 0.142	−0.223 ± 0.098
Clipping	0.925 ± 0.140	−0.032 ± 0.069
Coiling	0.832 ± 0.128	−0.074 ± 0.072

**Table 6:** Odds Ratios and Standardized Logistic Regression Coefficients (all SAH patients). Values are presented as mean ± standard deviation across test folds from repeated cross-validation.

Feature	Odds Ratio	Standardized Coefficient
Age at SAH	0.960 ± 0.019	−0.546 ± 0.259
Hunt & Hess grade	1.244 ± 0.108	0.251 ± 0.105
Fisher grade	1.359 ± 0.166	0.240 ± 0.104
CNS infection	1.010 ± 0.108	0.001 ± 0.029
EVD implanted	1.577 ± 0.269	0.212 ± 0.089
Aneurysm	4.716 ± 3.801	0.588 ± 0.349
Aneurysm maximum diameter	0.975 ± 0.031	−0.102 ± 0.128
Aneurysm dome height	1.015 ± 0.015	0.050 ± 0.049
Female sex	1.007 ± 0.063	0.002 ± 0.030
ACom location	0.691 ± 0.176	−0.171 ± 0.103
Clipping	1.418 ± 0.370	0.091 ± 0.080
Coiling	1.355 ± 0.320	0.134 ± 0.133

## Data Availability

The data presented in this study are available on reasonable request from the corresponding author. The data are not publicly available due to privacy and ethical restrictions, as they contain sensitive patient information.

## Abbreviations

The following abbreviations are used in this manuscript:

SAH	spontaneous subarachnoid hemorrhage
aSAH	aneurysmal subarachnoid hemorrhage
EVD	external ventricular drainage
DALYs	disability-adjusted life years
DCI	delayed cerebral ischemia
WFNS	World Federation of Neurological Societies
ML	machine learning
CNS	central nervous system
CTA	computed tomography angiography
MRA	magnetic resonance angiography
DSA	digital subtraction angiography
TCD	transcranial Doppler
MCA	middle cerebral artery
SVMs	support vector machines
KNN	k-nearest neighbors
ACom	anterior communicating artery
SMOTE	Synthetic Minority Oversampling Technique
CV	cross-validation
AUC-ROC),	area under the receiver operating characteristic curve
AUC-PR	area under the precision–recall curve
AP	average precision
bACC	balanced accuracy
PPV	positive predictive value
NPV	negative predictive value
TPR	true positive rate
TNR	true negative rate
PR	precision–recall
MIT	Massachusetts Institute of Technology
ACA	Anterior Cerebral Artery
AICA	Anterior Inferior Cerebellar Artery
BA	Basilar Artery
ICA	Internal Carotid Artery
PCA	Posterior Cerebral Artery
PCom	Posterior Communicating Artery
PICA	Posterior Inferior Cerebellar Artery
SCA	Superior Cerebellar Artery
VA	Vertebral Artery
